# Clinically significant changes in burden and depression among dementia caregivers following nursing home admission

**DOI:** 10.1186/1741-7015-8-85

**Published:** 2010-12-17

**Authors:** Joseph E Gaugler, Mary S Mittelman, Kenneth Hepburn, Robert Newcomer

**Affiliations:** 1 School of Nursing, Center on Aging, University of Minnesota, Minneapolis, MN, USA; 2 Department of Psychiatry, New York University Langone Medical Center, New York, NY, USA; 3 School of Nursing, Emory University, Atlanta, GA, USA; 4 Department of Social and Behavioral Science, University of California, San Francisco, CA, USA

## Abstract

**Background:**

Although extensive research exists on informal long-term care, little work has examined the clinical significance of transitions in family caregiving due to a lack of established clinical cut-points on key measures. The objectives of this study were to determine whether clinically significant changes in symptoms of burden and depression occur among caregivers within 12 months of nursing home admission (NHA) of their relatives with dementia, and to identify key predictors of clinically persistent burden and depression in the first year after institutionalization.

**Methods:**

Secondary longitudinal analysis of dementia caregivers were recruited from eight catchment areas in the United States with 6- and 12-month post-placement follow-up data. The sample included data on 1,610 dementia caregivers with pre- and six-month post-placement data and 1,116 with pre-placement, six-month, and 12-month post-placement data. Burden was measured with a modified version of the Zarit Burden Inventory. Depressive symptoms were assessed with the Geriatric Depression Scale.

**Results:**

Chi-square analyses found significant (*P* < .05) reductions in the number of caregivers who reported clinically significant burden and depressive symptoms after NHA compared to pre-placement. Logistic regression models revealed that wives and daughters were most likely to experience clinically persistent burden and husbands were most likely to experience clinically significant depression after NHA.

**Conclusions:**

In addition to suggesting that clinically significant decreases in caregiver burden and depression are likely to occur following institutionalization, the results reveal particular subsets of caregivers who are at continued risk of distress. Such findings can facilitate development of screening processes to identify families at-risk following institutionalization.

## Background

Over 50 million individuals provide unpaid care to adults who are disabled or ill http://www.caregiver.org. The prominence of informal care in the U.S. has led to a large number of studies describing family caregivers [1], examining stress in family caregiving [2,3], and evaluating the effectiveness of psychosocial interventions and respite services for caregiving families [4-6]. As chronic illnesses such as Alzheimer's disease or other dementias progress, critical health transitions (diagnosis, institutionalization, bereavement) may exacerbate negative health outcomes for persons with dementia or their family caregivers. Nursing home admission (NHA) in particular is a transition that is considered a key clinical marker of dementia progression [7].

Although extensive research exists on informal long-term care, little work has examined the clinical significance of transitions or other phenomena due to a lack of established clinical cut-points on key caregiving measures [8]. This has made it difficult to interpret results or effects of interventions for dementia caregivers. The high prevalence of dementia among nursing home residents (69% of all nursing home residents suffer from some form of cognitive impairment) [9] may place ongoing care demands onto family members. Several studies have noted that families continue to provide various types of informal (that is, unpaid) assistance to relatives in nursing homes (NHs). Such assistance ranges from visits, to care provision, to staff interaction [10-12]. For these reasons, it would be valuable to determine whether NHA has a clinically significant impact on caregiving outcomes and to identify those variables that predict clinically significant levels of burden and depression among caregivers after NHA. Using a large, multi-regional, and longitudinal data set of dementia family caregivers, the present study sought to: 1) determine whether clinically significant reports of burden or depressive symptoms change within 12 months of NHA, and 2) identify factors prior to NHA that predict clinically persistent burden and depression within the first year following institutionalization. These insights will serve to guide the development of effective intervention strategies that offer psychosocial support to dementia caregivers grappling with the potential challenges of NHA.

### Caregiving and institutionalization

Systematic reviews have noted that caregiving burden (or feelings of being overwhelmed with various facets of care), emotional fatigue, and perceptions of entrapment in the caregiving role are at least as important as care recipients' functional and cognitive decline in predicting risk of NH entry [13]. Longitudinal analyses of dementia caregiving make clear that caregiving does not "end" with the institutionalization of a cognitively impaired elderly relative [14]. Instead, family members remained engaged in the lives of institutionalized relatives. While the provision of "hands-on" technical care such as ambulation and transferring are often assumed by direct care workers in the NH setting, family involvement continues and ranges from regular visits, to ongoing provision of more instrumental forms of direct care (such as transportation and financial management), to interaction with staff to ensure proper care is delivered [12,15-18]. Some studies have found that various measures of caregiving stress or depressive symptoms remain stable, or in some cases, increase with NHA [19-23]. Contradictory findings from other studies suggest that institutionalization may result in decreased stress or depressive symptoms following NH placement as well as improved physical health for caregivers (for example, somatic symptoms, biomarkers of cardiovascular health) [20,24-27]. Some reasons for these discrepant findings include the fairly small samples included in prior research studies, which may have limited the statistical power necessary to detect significant changes in key caregiving outcomes across the NH transition. Similarly, variation in when caregivers are assessed prior to and after NHA (that is, length of follow-up) may have also obscured both the short- and long-term implications of institutionalization on dementia caregivers' stress, depressive symptoms, or other dependent variables.

Studies of dementia caregiving have identified several predictors of depressive symptoms and burden following institutionalization. Behavior problems of care recipients prior to or at the time of NHA appear to predict greater caregiver burden and reduced family involvement following NH placement (for example, visits, care provision) [22,28,29]. Spouse caregivers appear more likely to report greater burden, depressive symptoms, and dissatisfaction after NHA than adult child or other caregivers [21,30]. Wives in particular tend to invest greater emotional resources in their roles as "caregivers," and thus may be less willing to relinquish day-to-day care responsibility to a NH and are more involved in care delivery and supervision when compared to other types of caregivers [2,12,31,32]. Family caregivers' lack of satisfaction with help received from others [21,33], the increased functional or cognitive impairment of the relative [33,34], and less involvement with the relative following NHA [34] also appear to be associated with greater distress in caregivers after institutionalization.

### Research focus

Most caregiving research has failed to identify the clinical significance of reported results [8]. Many measures of caregiver stress or well-being do not have established cut-points signifying the presence of clinically relevant symptoms. This has made it difficult to interpret empirical associations between predictor variables and outcomes or determine whether an intervention has had a clinically meaningful effect. The present study advances current research on dementia caregiving and NHA in two ways. First, we developed clinically significant cut-points and analyzed data on the prevalence of clinically significant burden and depression prior to and up to 6- and 12-months post-placement. This initial analysis made it possible to estimate the proportion of caregivers who experienced clinically significant burden and depression in the months following NHA. Given the size of the sample and the amount of longitudinal, post-placement data available, this study aimed to reconcile conflicting results on changes in key caregiving outcomes prior to and following NHA. The second objective was to determine what pre-placement factors predicted clinically persistent burden and depressive symptoms among caregivers up to one year following institutionalization. Variables considered in the predictive analysis included those shown in previous studies to predict NHA in dementia and indicators derived from post-placement analyses of caregiver depression and stress [21,22,28,29,33,34]. Per other conceptualizations of stress in dementia caregiving [35], pre-placement burden was considered a predictor of post-placement depressive symptoms, as emotional appraisals of dementia care demands (that is, burden) are postulated to influence more global psychological outcomes (that is, depression). Since it is considered a global outcome of dementia caregiving, pre-placement depressive symptoms were not considered as a predictor of post-placement burden in subsequent analyses.

This study extends our previous research examining changes in caregiver burden and depressive symptoms after institutionalization. In a prior study [31] we used the full 6- and 12-month post-placement cohorts of the data set reported here to examine trajectories of change in burden in depressive symptoms prior to and up to one year after NHA. This previous work noted that the amount of change prior to and after institutionalization appeared to drop below clinical thresholds of burden 6- and 12-months following NHA. These intriguing initial findings led to the analyses reported in this study, which is a comprehensive, more focused analysis of clinically relevant change in burden and depressive symptoms after the NH transition. More specifically, this study identifies those factors that resulted in *clinically *persistent levels of burden and depression prior to and up to one year following NHA when compared to those who fell below these clinical thresholds (thus resulting in a more interpretable comparison). For these reasons the current study builds on our prior work and serves as an independent, notable contribution to understanding how families adapt emotionally and psychologically to NHA. Specifically, understanding factors that are linked to ongoing and clinically relevant burden and depressive symptoms during institutionalization could help social workers, nursing staff (for example, directors of nursing, registered nurses, certified nurse assistants), and medical directors to identify caregivers who are in need of support during NH entry, thus helping to facilitate families' adaptations to a relative's institutionalization.

## Methods

### The Medicare Alzheimer's Disease Demonstration Evaluation

The Medicare Alzheimer's Disease Demonstration Evaluation (MADDE) was a randomized controlled evaluation of a combined case management and Medicare-reimbursed home care benefit service that was implemented in eight communities in the U.S. A detailed description of MADDE is provided in a supplemental issue of *Health Services Research *[36]. Briefly, to be eligible for MADDE, care recipients (a) had a physician-certified diagnosis of an irreversible dementia, (b) were enrolled or eligible for Parts A and B of Medicare, (c) had service needs, and (d) resided at home at baseline in one of the eight MADDE catchment areas. After 60 days in a NH, MADDE service benefits were terminated. Nursing home admissions for less than 60 days that resulted in community discharge were recorded in MADDE but were not counted as "permanent" institutionalization episodes [37]. The caregiver was defined as the relative who provided the most help to the person with dementia. The baseline interview was in-person and biannual follow-up interviews were conducted via telephone over a three-year period. Interviews were administered up to 12 months following NHA. This study received Institutional Review Board exempt approval from the University of Minnesota Human Subjects Committee (IRB# 0611E96989).

### Sample

The baseline sample of MADDE included 5,831 caregivers and care recipients with dementia. Over 40% (43.9%) of the persons with dementia were institutionalized during the three-year follow-up period. MADDE collected data on 1,610 caregivers six months after NHA (the six-month post-placement panel) and 1,116 caregivers 12 months following NH entry (the 12-month post-placement panel). The pre-placement interview was the last interview conducted prior to NHA. Baseline and outcomes data are presented in Table 1 for the 6- and 12-month post-placement panels.

**Table 1:** Descriptive Information, 6-month (N = 1,610) and 12-month (1,116) Post-Placement Cohorts.

	6-Month Post-Placement Cohort			12-Month Post-Placement Cohort		
	***M/%***	***SD***	***Range***	***M/%***	***SD***	***Range***

*Context of Care*						
						
Site						
Florida	8.9%			7.6%		
Illinois	15.9%			16.8%		
Minnesota	21.9%			22.0%		
New York	13.2%			14.4%		
Ohio	14.7%			16.2%		
Oregon	9.6%			8.4%		
Tennessee	9.6%			8.3%		
West Virginia	6.1%			6.2%		
						
CR is female	59.3%			63.7%		
						
CR is Caucasian	92.0%			92.2%		
						
CR age (years)	71.47	7.62	30.63 to 102.10	78.83	7.41	47.03 to 97.06
						
CR is Medicaid eligible	50.3%			55.1%		
						
CR lives with CG	73.7%			72.5%		
						
Treatment	51.0%			52.2%		
						
Kin Relationship						
Wife	33.4%			29.0%		
Husband	17.8%			19.1%		
Daughter	26.6%			29.1%		
Other	22.2%			22.8%		
						
CG age (years)	63.56	14.42	23.00 to 100.00	63.01	14.31	25.00 to 100.00
						
CG income (1 = under $4,999; 11 = $50,000 and above)	5.81	2.91	1.00 to 11.00	5.90	2.91	1.00 to 11.00
						
CG is employed	33.1%			33.8%		
						
CG education (1 = elementary school; 6 = post-graduate)	3.54	1.33	0.00 to 6.00	3.53	1.33	0.00 to 6.00
						
Duration of care at baseline (in months)	44.21	38.53	0.00 to 360.00	43.98	39.09	0.00 to 360.00
						
Time to NHA from pre-placement (days)	113.86	69.80	0.00 to 372.00	99.98	50.31	0.00 to 372.00
						
*Dementia Severity*						
						
MMSE at baseline	14.29	7.89	0.00 to 30.00	14.08	8.14	0.00 to 30.00
						
Pre-placement behavior problems	9.67	4.06	0.00 to 19.00	9.74	4.01	0.00 to 19.00
						
*Functional Impairment*						
						
Pre-placement ADLs	4.34	2.55	0.00 to 10.00	4.26	2.48	0.00 to 10.00
						
Pre-placement IADLs	7.13	1.20	0.00 to 8.00	7.10	1.20	1.00 to 8.00
						
Pre-placement caregiving hours (typical week)	74.67	61.43	0.00 to 988.00	74.66	68.03	0.00 to 988.00
						
Pre-placement unmet needs	2.92	4.31	0.00 to 18.00	3.02	4.36	0.00 to 18.00
						
*Resources*						
						
Pre-placement chore service use (times)	29.76	98.08	0.00 to 1300.00	30.44	107.52	0.00 to 1300.00
						
Pre-placement personal care use (times)	67.56	193.55	0.00 to 1456.00	77.26	210.83	00.00 to 1456.00
						
Pre-placement adult day service use (days)	19.74	37.06	0.00 to 120.00	20.74	38.00	0.00 to 120.00
						
Pre-placement overnight hospital use (times)	2.05	7.97	0.00 to 120.00	2.23	9.18	0.00 to 120.00
						
Pre-placement secondary caregiving hours (typical week)	6.42	20.97	0.00 to 168.00	7.73	23.42	0.00 to 168.00
						
CG pre-placement self-reported health (1 = excellent; 4 = poor)	2.05	.83	1.00 to 4.00	2.05	.82	1.00 to 4.00
						
CG pre-placement ADLs	.24	.69	0.00 to 5.00	.22	.63	0.00 to 5.00
						
CG pre-placement IADLs	.78	1.51	0.00 to 8.00	.74	1.48	0.00 to 8.00
						
*Outcomes*						
						
Pre-placement: Clinically significant burden^a^	52.9%			52.9%		
						
6-month post-placement: Clinically significant burden	28.3%			21.8%		
						
12-month post-placement: Clinically significant burden	-			16.7%		
						
Pre-placement: Clinically significant depression^b^	35.8%			36.2%		
						
6-month post-placement: Clinically significant depression	31.1%			25.3%		
						
12-month post-placement: Clinically significant depression	-			27.2%		
						
Persistent burden^c^	22.1%			9.1%		
						
Persistent depression^c^	20.9%			12.4%		

NOTE: *M *= mean; *SD *= standard deviation; CR = care recipient; CG = caregiver; NHA = nursing home admission; ADL = activities of daily living; IADLs = instrumental activities of daily living; MMSE = Mini-Mental State Examination

^a^Those caregivers who indicated a Zarit Burden Inventory score of 13.00 or higher

^b^Those caregivers who reported a score of greater than or equal to six on the Geriatric Depression Scale

^c^Caregivers who remained in the clinically high burden and depression categories prior to and in the 6- and 12-month intervals following placement

## Measures

### Outcomes

The outcome variables were caregiver depressive symptoms, measured with the 15-item Geriatric Depression Scale (GDS) [38] and caregiver burden, measured with a 7-item short form of the Zarit Burden Interview (ZBI) [39]. Based on prior sensitivity and specificity analyses on the 15-item version of the GDS [40,41], a score of greater than or equal to six on the GDS was used to identify dementia caregivers suffering from *clinically significant depression*.

A clinically established cut-off for burden is less clear. For these reasons, we calculated a receiver operating characteristic (ROC) curve using the full baseline sample of MADDE (*N *= 5,831) to develop a potential cut-off score at which sensitivity and specificity (or the true negative rate) for identifying the clinically established cut-off for depression described above were optimized [31]. A cut-off score of 13.50 on the ZBI (range = 0.00 to 28.00) was found to simultaneously maximize the sensitivity (correctly identifying 77% of the cases with depression) and specificity (identifying 71% who do not have depression) of the GDS. The area under the ROC curve was a high proportion of the overall space (.814) [42]. In subsequent analyses caregivers who reported a ZBI score of 13.00 or higher (as non-integer summed scores are not possible on the ZBI) were considered to have a c*linically significant burden*.

A second objective of the current study was to identify predictors of persistently high depression or burden following the institutionalization transition. *Persistent burden *and *persistent depression *were operationalized as remaining at or above the clinical significance cut-points of burden and depression at the pre- and post-placement assessment intervals.

### Dementia severity

Case managers administered the Mini-Mental State Examination (MMSE) [43] to care recipients at baseline to measure cognitive status. Frequency of care recipient behavior problems was assessed with the Memory and Behavior Problems Checklist, completed by caregivers at pre-placement [44].

### Functional impairment

Measures of care recipients' pre-placement functional status included dependence in activity of daily living tasks (ADLs) [45] and instrumental activity of daily living tasks (IADLs) [46]. After caregivers reported whether care recipients needed no help, some help, or maximum help to perform each ADL or IADL, caregivers then indicated whether the care recipient was receiving enough help for that particular ADL or IADL (yes = 1; no = 0). These unmet need items for each ADL or IADL task was then summed at pre-placement [47]. The number of hours caregivers typically spent providing assistance to care recipients was also included at pre-placement (primary informal caregiving hours).

### Resources

Three community-based services (chore, personal care, and adult day care) and overnight hospital use for the care recipient were reported by caregivers at pre-placement. Secondary caregiving hours were measured at pre-placement by asking respondents how many hours they typically received help from other family members or friends in providing assistance to the care recipient (secondary caregiving hours). Other resource variables considered at pre-placement included caregivers' self-reported health status and caregivers' own functional dependency as measured by five ADLs and eight IADLs.

### Analysis

#### Objective 1: Description of clinically significant burden and depression across NHA

The first analysis examined changes in clinically significant burden and depression at the pre- and post-placement intervals. Chi-square analyses were conducted to determine whether there were statistically significant changes (*P* < .05) in the percentage of caregivers who reported clinically significant burden at pre-placement compared to caregivers at the 6-month and 12-month post-placement interviews. A parallel set of chi-square analyses were conducted to examine changes in clinically significant depression.

#### Objective 2: Predicting persistent burden and depression across NHA

The second objective was to identify those pre-placement variables that accounted for persistent burden or depressive symptoms in the 6- and 12-month post-placement panels. We used separate logistic regression models to identify variables that were predictive of persistent burden or depression up to 6 and 12 months after NHA using all of the variables described above as predictors. In order to ensure a relevant comparison, the reference category consisted of caregivers who scored below the clinical threshold of burden or depression at pre-and post-placement. All background/contextual, dementia severity, functional impairment, and resource indicators were included as independent variables. Since conceptual models of dementia caregiving often position emotional stressors as predictors of global outcomes such as depression [35], pre-placement burden was included as an additional predictor of persistent depressive symptoms in the logistic regression models.

## Results

### Objective 1: Description of clinically significant burden and depressive symptoms across the placement transition

The percentage of caregivers who reported clinically significant burden decreased from pre-placement to the six-month post-placement follow-up (pre-placement burden = 52.9%, six-month post-placement burden = 28.3%; *df = *1*; χ^2 ^= *162.30*; P* < .001). Although statistically significant, the reduction in clinically significant depressive symptoms up to six months after NHA was less pronounced (pre-placement depressive symptoms = 35.8%, six-month post-placement depressive symptoms = 31.1%; *df = *1*; χ^2 ^= *315.65*; P* < .001). Similar findings occurred in the 12-month post-placement panel. The percentage of caregivers who indicated clinically significant burden dropped considerably from pre-placement to the 12-month post-placement interview (pre-placement burden = 52.9%, 12-month post-placement burden = 16.7%; *df = *1*; χ^2 ^= *92.18*; P* < .001). Significant, although smaller, reductions also occurred in reports of clinically significant depression up to one year after NHA (pre-placement depressive symptoms = 36.2%; 12-month post-placement depressive symptoms = 27.2%; *df = *1*; χ^2 ^= *191.66*; P* < .001).

### Objective 2: Predicting persistent burden and depression across NHA

#### Persistent burden

Table 2 provides the results of the logistic regression models predicting persistent burden up to 6 and 12 months post-placement, respectively. The models accounted for 49% of the variance in the 6-month post-placement panel and 63% of the variance in the 12-month post-placement panel (Nagelkerke *R^2 ^*= .49 and .63, respectively). Several variables were significantly related (*P* < .05) to persistent burden across the post-placement panels, including site (caregivers from Florida or Oregon), race/ethnicity (Caucasian), employment, longer duration of care, more time spent in at-home care, greater unmet need, and more frequent behavior problems. With each point increase in subjective health impairment, caregivers were 2.36 and 3.01 times more likely to report persistent burden in the 6- and 12-month post-placement panels, respectively. Gender and kin relationship in particular appeared to have a strong influence on persistent burden; when compared to other caregivers, women reported more persistent burden than men. Wives were over eight times more likely to experience persistent burden in the 6-month post-placement panel (*OR *= 8.84) and even more likely to do so in the 12-month post-placement panel (*OR *= 29.16). Daughters also appeared more than twice as likely to indicate persistent burden when compared to other caregivers in the 6-month (*OR *= 2.75) and 12-month post-placement panels (*OR *= 3.89).

**Table 2:** Predictors of persistent burden in the 6-month and 12-month Post-Placement Panels (N = 1,015; 560 respectively)a

	*6-Month Post-Placement Panel*			*12-Month Post-Placement Panel*		
***Variables***	***B***	***SE***	***OR***	***B***	***SE***	***OR***

*Context of Care*						
Site						
Florida	1.85***	.44	6.36	2.05***	.81	7.78
Illinois	-.12	.40	.77	-.32	.70	.73
Minnesota	.00	.39	1.00	-.31	.68	.73
New York	.57	.42	1.76	-1.62	1.01	.20
Ohio	.51	.39	1.66	.75	.66	2.11
Oregon	1.11*	.44	3.02	1.87*	.78	6.51
Tennessee	.32	.43	1.38	.10	.80	1.10
West Virginia (reference)						
						
CR is female	.26	.37	1.30	-.64	.67	.53
						
CR is Caucasian	.65*	.33	1.92	2.28**	.75	9.77
						
CR age	-.01	.01	.99	-.01	.03	.99
						
CR is Medicaid eligible	.19	.19	1.21	.50	.39	1.65
						
CR lives with CG	.23	.27	1.26	.14	.62	1.15
						
Treatment	-.10	.18	.91	-.42	.36	.66
						
Kin relationship						
Wife	2.18***	.49	8.84	3.37**	1.01	29.16
Husband	.77	.43	2.15	2.33*	.96	10.26
Daughter	1.01***	.28	2.75	1.36*	.57	3.89
Other (reference)						
						
CG age	-.01	.01	.99	-.03	.03	.97
						
CG income	.00	.04	1.00	.09	.08	1.10
						
CG is employed	.59*	.24	1.81	1.13*	.46	3.10
						
CG education	.11	.08	1.12	-.29*	.15	.75
						
Duration of care	.01**	.00	1.01	.01**	.00	1.01
						
Time to NHA from pre-placement						
0 to 61 days (reference)						
61.01 to 105 days	-.13	.26	.87	-.53	.49	.59
105.01 to 151 days	-.01	.25	.99	-.63	.50	.53
Over 151 days	.19	.25	1.21	-.22	.47	.81
						
*Dementia severity*						
						
MMSE at baseline	.01	.01	1.01	.05	.02	1.05
						
Pre-placement behavior problems	.13***	.03	1.14	.24***	.05	1.28
						
*Functional impairment*						
						
Pre-placement ADLs	-.04	.05	.96	.12	.10	1.13
						
Pre-placement IADLs	.05	.10	1.05	-.07	.22	.94
						
Pre-placement caregiving Hours						
0 to 20 hours (reference)						
20.01 to 70 hours	1.20***	.27	3.32	1.11*	.56	3.05
70.01 hours to 118 hours	1.58***	.30	4.83	1.77**	.63	5.85
Over 118 hours	1.57***	.31	4.81	2.51***	.59	12.28
						
Pre-placement unmet needs	.13***	.02	1.14	.18***	.05	1.20
						
*Resources*						
						
Any pre-placement chore service use	.15	.20	1.16	.11	.40	1.12
						
Any pre-placement personal care use	.09	.21	1.09	-.04	.41	.96
						
Any pre-placement adult day service use	-.08	.20	.93	-.42	.40	.66
						
Any pre-placement overnight hospital use	.08	.22	1.09	.17	.43	1.19
						
Any pre-placement secondary caregiving hours	-.17	.18	.84	-1.14**	.36	.32
						
CG pre-placement self-reported health	.86***	.13	2.36	1.10***	.25	3.01
						
CG pre-placement ADLs	-.23	.15	.79	.10	.28	1.10
						
CG pre-placement IADLs	.08	.08	1.08	-.09	.15	.91

^a^Caregivers who did not indicate clinically significant burden at pre-placement or post-placement intervals was the reference category (*n *= 659 in 6-month post-placement panel; *n *= 459 in the 12-month post-placement panel)

NOTE: CR = care recipient; CG = caregiver; NHA = nursing home admission; ADL = activities of daily living; IADLs = instrumental activities of daily living; MMSE = Mini-Mental State Examination

#### Persistent depression

Table 3 presents the results of the logistic regression models predicting persistently high symptoms of depression in the 6- and 12-month post-placement panels. The post-placement models accounted for a considerable amount of variance in persistent depression in the 6-month (Nagelkerke *R^2 ^*= .64) and 12-month (Nagelkerke *R^2 ^*= .71) post-placement panels. Husbands were 4.87 times and 5.89 times more likely to indicate persistent depression than other caregivers in the 6- and 12-month post-placement panels, respectively. Caregivers who reported a point increase in subjective health impairment were 2.11 and 2.39 times more likely to indicate persistent depression in the 6- and 12-month post-placement panels, respectively. In addition, caregivers in New York were less likely to indicate persistent depression in the 6-month and 12-month post-placement panels (*OR *= .33; *OR *= .08, respectively).

**Table 3:** Predictors of persistent depression in the 6-month and 12-month Post-Placement Panels (N = 1,208; 710 respectively)a

	*6-Month Post-Placement Panel*			*12-Month Post-Placement Panel*		
***Variables***	***B***	***SE***	***OR***	***B***	***SE***	***OR***

*Context of Care*						
Site						
Florida	.59	.53	1.80	-.70	.83	.50
Illinois	-.46	.50	.63	-1.21	.78	.30
Minnesota	-.90	.49	.41	-2.15**	.79	.12
New York	-1.11*	.53	.33	-2.50**	.89	.08
Ohio	-.32	.50	.73	-1.43	.77	.24
Oregon	-.17	.55	.84	-.51	.94	.60
Tennessee	-.20	.53	.82	-.85	.81	.43
West Virginia (reference)						
						
CR is female	-.94*	.41	.39	-1.11	.70	.33
						
CR is Caucasian	.24	.39	1.27	.03	.74	1.03
						
CR age	-.03	.02	.97	-.05	.03	.95
						
CR is Medicaid eligible	.24	.21	1.27	.29	.36	1.34
						
CR lives with CG	.19	.31	1.21	.35	.59	1.41
						
Treatment	.12	.20	1.13	.01	.34	1.01
						
Kin relationship						
Wife	.51	.51	1.66	1.18	.92	3.26
Husband	1.58**	.49	4.87	1.77*	.84	5.89
Daughter	.34	.34	1.41	1.00	.70	2.72
Other (reference)						
						
CG age	.01	.02	1.01	.02	.03	1.02
						
CG income	-.08*	.04	.92	-.07	.07	.94
						
CG is employed	.23	.28	1.26	-.24	.45	.59
						
CG education	-.08	.08	.92	-.22	.14	.81
						
Duration of care	.00	.00	1.00	.01	.00	1.01
						
Time to NHA from pre-placement						
0 to 63 days (reference)						
63.01 to 99.98 days	.08	.28	1.08	.64	.49	1.89
99.99 to 140.75 days	.22	.27	1.24	.78	.45	2.19
Over 140.75 days	.26	.31	1.30	.69	.48	1.99
						
*Dementia severity*						
						
MMSE at baseline	-.02	.01	.98	-.03	.02	.97
						
Pre-placement behavior problems	.01	.03	1.01	.01	.05	1.01
						
*Functional impairment*						
						
Pre-placement ADLs	.01	.06	1.02	.02	.09	1.02
						
Pre-placement IADLs	-.08	.12	.93	-.14	.24	.87
						
Pre-placement caregiving hours						
0 to 20 hours (reference)						
20.01 to 70 hours	-.50	.32	.61	.64	.62	1.90
70.01 hours to 112 hours	-.33	.34	.72	.90	.67	2.47
Over 112 hours	-1.02**	.36	.36	-.15	.64	.86
						
Pre-placement unmet needs	.00	.03	1.00	.03	.04	1.03
						
*Resources*						
						
Any pre-placement chore service use	.35	.22	1.42	.32	.37	1.37
						
Any pre-placement personal care use	-.06	.24	.94	.28	.40	1.32
						
Any pre-placement adult day service use	-.22	.22	.80	-.34	.37	.72
						
Any pre-placement overnight hospital use	.36	.26	1.43	-.21	.47	.81
						
Pre-placement secondary caregiving hours (less than 1 hour per week is reference)	-.11	.21	.90	-.90**	.34	.41
						
CG pre-placement self-reported health	.74***	.14	2.11	.87***	.23	2.39
						
CG pre-placement ADLs	.26	.18	1.29	.34	.27	1.41
						
CG pre-placement IADLs	.14	.08	1.15	.34**	.13	1.40
						
Pre-placement burden	.28***	.02	1.32	.28***	.04	1.32

^a^Caregivers who did not indicate clinically significant depression at pre-placement or post-placement intervals was the reference category (*n *= 871 in the 6-month post-placement panel; *n *= 572 in the 12-month post-placement panel)

NOTE: CR = care recipient; CG = caregiver; NHA = nursing home admission; ADL = activities of daily living; IADLs = instrumental activities of daily living; MMSE = Mini-Mental State Examination

## Discussion

The proportion of caregivers who reported clinically significant levels of burden prior to placement was dramatically reduced 6 and 12 months following NHA. A similar change occurred in clinically significant depression following institutionalization, although the reduction was less pronounced. Nursing homes assume many of the challenging care tasks that informal caregivers had provided. Specifically, NHs play a strong "substitution" role as formal care providers (staff) assume the care responsibilities formerly provided by informal caregivers (family members) [48,49]. This may lead to considerable emotional and psychological relief for family members.

This is not to say that some family caregivers do not experience distress or challenges during NHA. While several sociodemographic and contextual characteristics predicted persistent, clinically significant burden across the 6- and 12-month post-placement panels, kin relationship emerged as a potent influence. Female caregivers, and wives in particular, appeared far more likely to experience persistent burden following institutionalization. Female caregivers generally are more involved in day-to-day hands-on care provision and may also have a more difficult time relinquishing this role to 24-hour NH care staff; for example, wives tend to indicate more emotional investment in their caregiving roles when compared to other caregivers and also provide more direct care than husbands at pre-placement. This may continue after institutionalization (for example, supervising the care tasks performed by NH staff, playing an advocacy role for the institutionalized husband to ensure proper care is delivered in the NH) [2,12,31,32]. Therefore, stress may not abate with NHA for female caregivers who are heavily engaged in the emotional and direct, day-to-day care challenges associated with assisting a relative with dementia. Caregivers who indicated more frequent behavior problems in their relatives, provided more hands-on care, reported unmet needs pertaining to functional dependence of care recipients, and struggled with health or functional impairments of their own were also more likely to experience burden following NH placement. It may be that care recipients with more severe functional or behavioral impairments are more difficult for NH staff to manage successfully, so much so that their families feel compelled to remain involved in the care of institutionalized relatives and to interact regularly with NH staff to ensure proper care is delivered [10,11]. Such involvement is likely difficult to maintain when caregivers suffer from their own health impairments, thus leading to elevated burden after NHA.

Husbands, on the other hand, were particularly susceptible to post-placement depression across the 6- and 12-month panels. Husbands may have difficulty in adapting to and confronting the emotional loss of a partner and disruption to the role of husband in the context of institutional placement. Moreover, husbands may be less likely to have a reservoir of social support to rely on during NHA. These factors could lead to increased depressive symptomatology. We also found that caregivers with greater subjective health impairment and pre-placement burden were more likely to experience persistent depression across post-placement panels, which adds to the evidence suggesting that caregivers with physical or emotional challenges during at-home care may have continued psychological difficulty with the NH transition. It is possible that caregivers with emotional distress and health impairments may have felt increased guilt when they had to relinquish at-home care responsibilities due to these issues.

We have noted in our prior research that the provision of intensive, individualized consultation can help mitigate reports of burden or depressive symptoms among dementia caregivers [24]. It appears that the expanded case management model of MADDE did not exert a significant influence on caregivers' depressive symptomatology or burden during the NH transition. Consideration of the descriptive findings here as well as our prior clinical work reiterates what is currently best-practice in dementia caregiving intervention: multi-component psychosocial interventions that combine individualized consultation with family sessions and ongoing support often exert the greatest benefits in improving dementia caregiver outcomes, delaying NHA, or enhancing dementia caregivers' experiences across key transition points [6,50]. Alternatively, case management/referral models such as the MADDE approach tend to yield less consistent effects on such outcomes.

Although this analysis had at its disposal a large, multi-regional data set, there are several limitations that are important to consider. One is that there is no specific information about the type of care received in the NH by the care recipient, extent of family care provided to cognitively impaired care recipients following institutionalization, or post-placement measures of care recipients' dementia severity or functional status. In addition, MADDE data were collected in the early 1990 s. Since then a number of developments have occurred in long-term care, such as market availability and the emergence of assisted living, which may influence families' placement decisions. Although the MADDE data set is large, it did not rely on probability sampling techniques. Since to our knowledge no "gold standard" clinical rating of burden currently exists [51], we had to rely on the GDS to establish the clinically significant cut-point for burden. The sample size is also largely Caucasian. While this is expected due to lower NH placement rates frequently reported by African-American and Latino caregivers [52], such disparity may still influence generalizability. As noted by Jacobson and colleagues [53], the construct of clinical significance is best operationalized as "recovered," "improved but not recovered," and "still dysfunctional." In order to improve clarity of our interpretation, we limited the outcome to those who "recovered" and those who were "still dysfunctional" per the Jacobson classification. Inclusion of the third category (improved but not recovered) may have offered a fuller picture as to how each of these three potential outcomes occurred across the NH transition.

Several site effects were also apparent [[31], p. 394]. A limitation of MADDE was the lack of regional data to explain why site variations occurred in outcomes such as burden or depressive symptoms. Issues ranging from culture, to varying methods of MADDE implementation, to staffing ratios may have accounted for site-level variation in key variables. Historical events (Hurricane Andrew occurred in Florida in 1992) may have also influenced dementia caregivers' reports of distress during MADDE. Overall, the variations across site suggest that future multi-regional caregiving studies (descriptive or intervention-focused) must incorporate site data to explain the manifestation of key outcomes.

As the nature of family involvement may shift with residential care placement, it is possible that measures of caregiver stress require modification to better capture dimensions pertinent to the NH environment following institutionalization. As we have emphasized in prior research [24,31] future measures should approach NHA as a transitional event with a focus on pre-admission and post-placement factors. Key pre-admission variables could include how the decision to institutionalize came about, how helpful the NH was in facilitating the move, difficulty in finding an appropriate facility, and overall satisfaction with the institutionalization experience. During post-placement, greater attention to family caregivers' experiences with NHs such as family-staff interactions, types of family involvement, perceptions of collaboration with NH staff, and ratings of the philosophy of care (for example, family-centered emphasis) may improve measurement of the NH transition's influence on dementia caregivers.

## Conclusion

The findings of these analyses have several implications for future research and practice. Perhaps the most striking result is the apparent effect of NHA on clinical reports of burden and depressive symptoms following institutionalization. Our results emphasize that placement itself may provide relief and reductions in negative outcomes for dementia caregivers, particularly burden (in which we found a more considerable decrease than in depressive symptoms). Is it possible to reconcile our results with the ongoing focus of federal, state, and local support services to prevent NHA and save taxpayer-financed healthcare costs (for example, Medicaid in the U.S.), since this study shows that for many dementia caregivers placement may actually exert significant, clinical benefits? Perhaps the emphasis on preventing institutionalization is less appropriate than identifying the right time to make the placement decision or determining what types of support are optimal to reduce burden and depression during the period the person is cared for at home as well as during the NH transition [54]. More widespread implementation of effective psychosocial interventions to reduce caregiver burden and depression prior to the transition to NH placement can ameliorate the symptoms of burden and depression and increase the likelihood that a placement event is not premature, but instead occurs at an appropriate time. Nursing home admission should be viewed as a key transition faced in the course of dementia as opposed to a clinical endpoint.

While NHA may help to reduce overall burden and depressive symptoms in most caregivers, some may experience continued emotional and psychological distress well after institutionalization. The results of this study help to construct a profile of caregivers most at-risk for clinically high, persistent negative outcomes in the months after NHA. Wives, daughters, caregivers who have challenges meeting the needs of care recipients, and caregivers who have difficulty with their own health needs appear particularly susceptible to clinically persistent burden in the months immediately following NHA. Husbands and caregivers who experience health impairments and emotional stressors of their own prior to institutionalization appear most likely to suffer from clinically significant depression throughout the NH transition. Our findings can serve as an initial step in developing a screening process to identify families at-risk for burden or depression immediately prior and subsequent to institutionalization. Such families might benefit from psychosocial interventions (such as those provided by NH social workers or family nurse practitioners) during NHA to ease the transition and alleviate adverse outcomes following institutionalization. For example, using a transition "coach" during the pre-admission phase, the entry process, and in the months following admission could provide education regarding the NH environment and care policies, management of emotions and stress that may occur during the entry process (for example, guilt or grief), validation of families' decisions, and advocacy in facilitating interactions between NH care staff and administration. Such an approach may help families navigate the NH transition, and the development and testing of this or similar interventions could serve as a focus for future evaluation.

## Abbreviations

ADLs: activities of daily living; *df *: degrees of freedom; GDS: Geriatric Depression Scale; IADLs: instrumental activities of daily living; MADDE: Medicare Alzheimer's Disease Demonstration Evaluation; MMSE: Mini-Mental Status Examination; NH: nursing home; NHA: nursing home admission; ROC: receiver operating characteristic; U.S.: United States; ZBI: Zarit Burden Inventory

## Competing interests

The authors declare that they have no competing interests.

## Authors' contributions

JEG conceptualized the current study, conducted the analyses, and wrote all sections of the manuscript. MSM assisted JEG in conceptualizing the current study, assisted JEG in interpreting the analysis, and reviewed and revised all sections of the manuscript. KH reviewed all sections of the manuscript and provided feedback on the analytic plan. RN provided feedback on the analytic plan, assisted JEG in interpreting the results, and reviewed and revised the manuscript. All authors read and approved the final manuscript.

## Pre-publication history

The pre-publication history for this paper can be accessed here:

http://www.biomedcentral.com/1741-7015/8/85/prepub
